# Antiarrhythmic effects and mechanisms of sodium-glucose cotransporter 2 inhibitors: A mini review

**DOI:** 10.3389/fcvm.2022.915455

**Published:** 2022-08-08

**Authors:** Jinchun Wu, Yanmin Liu, Xiaojuan Wei, Xiaofei Zhang, Yi Ye, Wei Li, Xiaoling Su

**Affiliations:** ^1^Department of Cardiology, Qinghai Provincial People's Hospital, Xining, China; ^2^Graduate School of Qinghai University, Qinghai University, Xining, China

**Keywords:** Na^+^ homeostasis, Ca^2+^ homeostasis, Na^+^-H^+^ exchanger regulatory factor (NHERF)1/2, Na^+^-Ca^2+^ exchanger (NCX), ventricular arrhythmia (VAs), atrial fibrillation, arrhythmia, sodium-glucose cotransporter 2 inhibitor

## Abstract

Sodium-glucose cotransporter 2 inhibitors (SGLT2i) are a new type of oral hypoglycaemic agent with good cardiovascular protective effects. There are several lines of clinical evidence suggest that SGLT2i can significantly reduce the risks of heart failure, cardiovascular death, and delay the progression of chronic kidney disease. In addition, recent basic and clinical studies have also reported that SGLT2i also has good anti-arrhythmic effects. However, the exact mechanism is poorly understood. The aim of this review is to summarize recent clinical findings, studies of laboratory animals, and related study about this aspect of the antiarrhythmic effects of SGLT2i, to further explore its underlying mechanisms, safety, and prospects for clinical applications of it.

## Introduction

**Figure 1:**
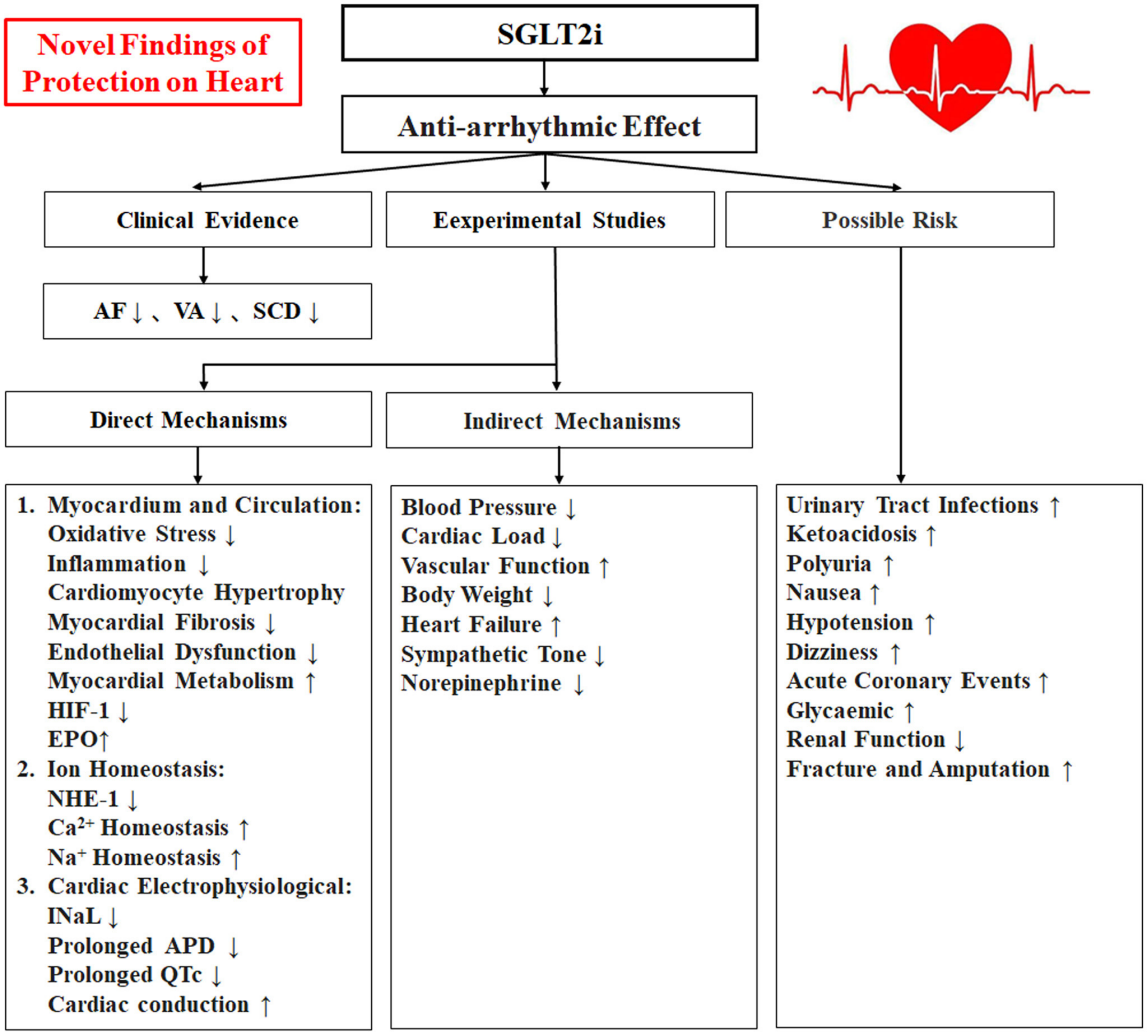
A schematic illustrating the relationship between the SGLT2i with antiarrhythmic effects and Mechanism. SGLT2i, Sodium-glucose cotransporter 2 inhibitors; AF, atrial fibrillation/atrial flutter; VA, ventricular arrhythmias; SCD, sudden cardiac death; HIF-1, hypoxia-inducible factor-1; EPO, erythropoietin; NHE-1, Na+-H+ exchanger 1; INaL, late Na+ current; APD, action potential duration.

Sodium-glucose cotransporter 2 inhibitors (SGLT2i) (including dapagliflozin, empagliflozin, sotagliflozin, and canagliflozin, among others) are novel oral hypoglycaemic agents with both cardiovascular and renal benefits that can significantly reduce hospitalization due to heart failure, decrease cardiovascular death, protect renal function and improve insulin resistance (1, 2). Recent clinical studies have shown that SGLT2i have anti-arrhythmic effects also (3, 4), and experimental studies have shown that SGLT2i may indirectly or directly affect on the onset of arrhythmias *via* alleviation of myocardium oxidative stress and inflammatory response, improvement of cardio fibrosis and endothelial dysfunction, promotion of cardiomyocyte energy and lipid metabolism, maintaining of cellular ion homeostasis, amelioration of electrophysiological remodeling, also improvement of heart failure, inhibition of cardiac sympathetic hyperinnervation and autonomic imbalance, reduction of body weight, through the above combined mechanisms to suppress arrhythmias (5, 6). This review we summarize the recent clinical evidence, studies of laboratory animals, and related studies about this aspect on the antiarrhythmic effects of SGLT2i, to further explore its underlying mechanisms, safety, and prospects for clinical applications of it (Figure 1).

## Clinical evidence of the antiarrhythmic effects of SGLT2i

### SGLT2i and atrial arrhuthmias

The DECLARE-TIMI 58 trial subgroup analysis in type 2 diabetes mellitus (T2DM) patients suggested that dapagliflozin reduced the risk of atrial fibrillation/atrial flutter (AF) events by 19% (HR: 0.81, 95% CI: 0.68–0.95, *p* = 0.009) (3). A meta-analysis showed that SGLT2i significantly reduced AF-related adverse events by 19.33% (RR: 0.83, 95% CI: 0.71–0.96, *p* = 0.01) (7). Another systematic review and meta-analysis indicated that SGLT2i was associated with a reduced risk of developing AF (RR: 0.82, 95% CI 0.70–0.96), however, there was no significant difference in reductions in the incidence of atrial flutter (RR: 0.83, 95% CI 0.58–1.17), and the occurrence of cardiac arrest (RR: 0.83, 95% CI 0.61–1.14) was not significantly different (8). More recently, an analysis of the large FDA adverse event reporting system reported that the diabetic patients treated with SGLT2i had a lower incidence of AF, which was highly indicated by its antiarrhythmic effect (9). However, although these were real-world data, the selected study patients were diagnosed with T2DM or cardiovascular diseases, which would expect a higher prevalence of AF (10), and it remains poorly understood if a potential beneficial SGLT2i effect on AF might be due to improving the heart failure, or whether it was the result from direct effect in the myocardium (11). Thus, further research on these issues is needed.

### SGLT2i and ventricular arrhythmias

The results of the DAPA-HF study indicated that dapagliflozin reduced the risk of ventricular arrhythmias (VA), cardiac arrest, or sudden death in patients with reduced ejection fraction heart failure (HR: 0.79, 95% CI 0.63–0.99, *p* = 0.037) (12). A meta-analysis showed that SGLT2i treatment also significantly reduced the risk of arrhythmias (OR: 0.81, 95% CI: 0.69–0.95, *p* = 0.008) and sudden cardiac death (SCD) outcomes (OR: 0.72, 95% CI: 0.54–0.97, *p* = 0.03) in patients with T2DM or heart failure (4). Another trial indicated that dapagliflozin reduced ventricular ectopic burden, and suggested it had an antiarrhythmic effect (13), however, there was also direct evidence decoding the effects of SGLT2i on VA in HF (14, 15). Meanwhile, there were no larger clinical research study results that explored the antiarrhythmic properties of SGLT2i in patients. Expectantly, several prospective studies had been performed, such as the empagliflozin -ICD trial will investigate the impact of empagliflozin on the burden of VA in patients with diabetes and an implanted implantable cardioverter-defibrillator (ICD) or cardiac resynchronization therapy (CRT) device (16). Thus, the mechanism of SGLT2i anti- VA is not entirely clear and needs further study.

## Direct mechanisms by which SGLT2i mediates anti-arrhythmic effects

Trigger and re-entrant were the two main and direct mechanisms of arrhythmogenesis, furthermore, the arrhythmias are more likely to be triggered when cells, hearts, or the whole-body system were subjected to pathological conditions, such as aggravated oxidative stress, activation the inflammation, acidosis, hypoxia, myocardial energy metabolism disturbance, microcirculation disorder, heart failure, sympathetic stimulation, etc. In brief, those were directly related to the occurrence of arrhythmias and can be considered as a direct role in the development of arrhythmias. The SGLT2i may act as an antiarrhythmic effect by inhibiting those conditions.

### The effect of SGLT2i on the myocardium oxidative stress and inflammatory response

Chronic systemic inflammation, oxidative stress, and fibrosis were closely linked, and those played a key role in the pathogenesis of arrhythmia occurrence (17). Treatment with antioxidants was shown to reduce cardiac pro-inflammatory and fibrotic markers (18, 19). A study reported that empagliflozin significantly reduced cardiomyocyte hypertrophy, and interstitial fibrosis, which indicated that empagliflozin reduction of cardiovascular oxidative stress and inflammation (20). In addition, it was reported that dapagliflozin administration led to a significant decrease in reactive oxygen and nitrogen species, as well as a significantly reduced myofibroblast infiltration and cardiac fibrosis in the myocardial infarction rat model (21), and dapagliflozin treatment significantly reduced cardiac NLRP-3 inflammasome activation, as well as inflammatory biomarkers along with antifibrotic effects in T2DM mice and mice (22–24). It was also shown that dapagliflozin decreased inflammatory cytokines in pigs with ejection fraction preserves heart failure, thereby improving cardiac function (25). It was also believed that canagliflozin had anti-inflammatory and antifibrotic properties, resulting in reduced levels of interleukin-6 (IL-6), tumor necrosis factor receptor-1 (TNF-1) in the serum (26). Beyond that, the high sensitivity of C-reactive protein was reported to be reduced by 54% after treated with empagliflozin in diabetes patients (27). Clinical studies have also shown that dapagliflozin significantly reduced the inflammatory response *in vivo* and decreased the incidence of adverse cardiovascular outcomes in patients after coronary interventional therapy (28). Thus, the evidence suggested that SGLT2i may act as an antiarrhythmic agent through anti-oxidative stress and anti-inflammatory responses.

### The effect of SGLT2i on the cardio fibroblasts and myocardial remodeling

Myocardial fibrosis was an integral part of cardiac remodeling, which led to a decline in cardiac function, even heart failure. Myocardium with abnormally activated fibroblasts secretes extracellular matrix proteins, resulting in impaired ventricular function and contractile dysfunction, promoting cardiac fibrosis, and causing arrhythmias eventually (29). Lee et al. (21) showed that dapagliflozin significantly inhibited cardiac fibrosis in post-myocardial infarction rat models. In addition, Kang et al. (30) provided that empagliflozin suppressed pro-fibrotic markers such as type I collagen, a-smooth muscle actin, connective tissue growth factor, and matrix metalloproteinase 2, and reduced TFG-β1 induced fibroblasts activation. In addition, SGLT2i significantly attenuated TGF-β-induced fibroblast activation, reduced myocardial fibrosis and myocardial remodeling, and further improved cardiac function (31). Therefore, this would be a potential anti-arrhythmic effects mechanism of SGLT2i.

### The effect of SGLT2i on the myocardium endothelial cells and endothelial dysfunction

A dysfunctional endothelium was defined as an imbalance between its integrity and function, which associated with a diminished vasodilatory capacity, inflammation, and prothrombosis, additionally, SGLT2i had a positive effect on the suppression of arrhythmia occurrence by improving endothelial dysfunction. Recently study demonstrated that in both human arterial coronary endothelial cells and human umbilical vein endothelial cells, empagliflozin inhibited the activity of the Na^+^-H^+^ exchanger 1 (NHE-1) activity (32), dapagliflozin decreased the LPS-induced increase in NHE-1 mRNA in cardio fibroblasts (33). It also reported that dapagliflozin significantly ameliorated peripheral microvascular endothelial dysfunction (34). Furthermore, empagliflozin has also been shown to reduce carotid radial pulse wave velocity and augment radial, carotid, and aortic arterial stiffness (35). Another study recently confirmed the positive effects of empagliflozin on endothelial function in patients with T2DM (36). Meanwhile, the underlying causes for endothelial dysfunction were varied, the process may involve oxidative stress and chronic inflammation (37, 38). This suggested that SGLT2i may act as an antiarrhythmic agent by protecting the endothelium's normal function.

### The effect of SGLT2i on the myocardium metabolic alteration

Under physiologic conditions, myocardial energy was mainly supplied by mitochondrial oxidative metabolism and glucose metabolism when myocardial energy metabolism changed and also promoted arrhythmogenesis (39–41). A study revealed that empagliflozin treatment by reducing triglyceride accumulation, significantly reduced myocardial and liver steatosis, it was not clear, however, whether the observed empagliflozin effect on cardiac triglyceride accumulation was tissue-specific (42, 43). A possible explanation for SGLT2i inhibition-mediated cardioprotection was ketone body formation (44), through stabilization of membrane potential, ketones increased mitochondrial biogenesis and exerted anti-arrhythmic effects (45). There was evidence that SGLT2i had a direct impact on the reduction of plasma glucose levels and shifting myocardial metabolism to fatty acid (46). In addition, A study suggested that dapagliflozin reduces hypoxia-inducible factor-1 (HIF-1) production, enhancing erythropoiesis by increasing erythropoietin (EPO) secretion and increasing myocardial oxygen supply and metabolic capacity (47). Based on the above research, According to current thinking, cardiac cell metabolism improvements are primarily responsible for SGLT2i's ability to inhibit arrhythmias, as well as reduction of cardiac cell necrosis and cardiac fibrosis (48, 49). Although the results described above were tempting, Further investigation is needed to elucidate SGLT2i's beneficial effects on cardiac metabolism and bioenergetics, as well as how to further exert the antiarrhythmic effect.

### The effect of SGLT2i on ion homeostasis in cardiomyocytes

Myocardial Ca^2+^ and Na^+^ homeostasis were essential for cardiac signal transduction, heart rhythm regulation, and cardiac myocyte energy production (50, 51), therefore, it is critical to study the molecular mechanisms involving Ca^2+^ and Na^+^ homeostasis to better understand the Mechanism of arrhythmia occurrence.

#### The effect of SGLT2i on Ca^2+^ handling in cardonmyocytes

Abnormal cardiomyocyte Ca^2+^([Ca^2+^]_c_) was one of the biological markers for the development of heart failure and death due to cardiovascular causes and was also responsible for arrhythmogenesis (52). Mustroph et al. (6) showed that empagliflozin reduced calcium/calmodulin-dependent protein kinase II (CaMK II) activity and CaMK II-dependent ryanodine receptor phosphorylation in the cardiomyocytes of mice with heart failure model; empagliflozin also reduced the human cardiomyocyte Ca^2+^ spark (CaS) frequency but increased the sarcoplasmic reticulum Ca^2+^ ([Ca^2+^]_SR_) levels and Ca^2+^ transient (CaT) amplitude, whereas CaMK II overexpression and Ca^2+^-dependent activation were the main causes of arrhythmogenesis. David et al. (53) showed that sotagliflozin improved left atrial structural remodeling and atrial cardiomyopathy-associated arrhythmias in rats with heart failure and that the main mechanism involved improving [Ca^2+^]_c_ handling in atrial cardiomyocytes. Lee et al. (54) showed that empagliflozin could block S2808 phosphorylation of ryanodine receptor (RyR) and increase sarco/endoplasmic reticulum Ca^2+^-ATPase 2a (SERCA2a) expression, which in turn improved Ca^2+^ homeostatic imbalances in ventricular myocytes, reduced the CaS frequency, increased the CaT amplitude, and increased [Ca^2+^]_SR_. It has been suggested that CaMK II can also stimulate the activity of NHE-1, and the downregulation of CaMK II activity after SGLT2i intervention may also inhibit NHE-1 activity (55). Hamouda et al. (56) showed that dapagliflozin could reduce the amplitude of CaT and L-type Ca^2+^ ([Ca^2+^]_L_) currents in diabetic rat cardiomyocytes, and [Ca^2+^]_L_ currents were the main trigger for [Ca^2+^]_SR_ release (57), Other studies also suggested that dapagliflozin inhibited arrhythmias by partially inhibiting [Ca^2+^]_L_ currents, which in turn inhibits [Ca^2+^]_SR_ release (58). In short, it was currently believed that SGLT2i can reduce myocardial cardiomyocyte Na^+^([Na^+^]_c_) and [Ca^2+^]_c_ concentrations, increase mitochondrial Ca^2+^ ([Ca^2+^]_m_) concentrations through NHE-1, and improve the expression of Ca^2+^ handling-related proteins or regulate myocardial [Ca^2+^]_c_ homeostasis to protect cardiac function and reduce arrhythmia occurrences, but the exact mechanisms need to be further explored.

#### The effect of SGLT2i on Na^±^ homeostasis in cardonmyocytes

The CAST study (Cardiac Arrhythmia Suppressing Trial) showed that common Na^+^ channel blockers had arrhythmogenic effects mainly by blocking the fast Na^+^ current (INa) (59); however, inhibiting the endogenous late Na^+^ current (INaL) elicited antiarrhythmic effects on hearts (60). SGLT2i rapidly reduced [Na^+^]_c_ overload in cardiomyocytes without blocking INa and thus may have antiarrhythmic effects (61). Studies had shown that SGLT2i affected myocardial [Na^+^]_c_ load by inhibiting the upregulation of NHE-1 during heart failure, which in turn reduced myocardial [Ca^2+^]_c_ load and decreased secondary myocardial membrane and mitochondrial Na^+^-Ca^2+^ exchangers, decreasing [Ca^2+^]_c_ concentrations and improving myocardial excitation-contraction coupling, including inhibiting arrhythmias (62). Uthman et al. (63) observed that empagliflozin inhibited NHE-1 and rapidly reduced myocardial [Na^+^]_c_ concentrations within the therapeutic range. It was also reported that empagliflozin had SGLT2i-independent activity and directly inhibited cardiac NHE-1, reducing myocardial [Na^+^]_c_ and [Ca^2+^]_c_ and increasing cardiomyocyte [Ca^2+^]_m_ (58). A study in mice with heart failure demonstrated that empagliflozin significantly reduced INaL but had no effect on INa, suggesting that empagliflozin had an antiarrhythmic effect (64). Some studies have suggested that SGLT2i had off-target effects; empagliflozin had no inhibition effects on NHX1 but reduced myocardial [Na^+^]_c_ and was independent of the range of intervention concentrations (65). It had been suggested that sodium-myoinositol cotransporter 1 (SMIT1), an SGLT isomeric structure expressed in the myocardium, which was overexpressed will further activate NADPH oxidase 2 (NOX2), and trigger myocardial [Na^+^]_c_ overload by increasing glucose uptake while enhancing the oxidative stress response, suggesting that SGLT2i may act in the same way to ultimately reduce [Na^+^]_c_ overload (66). It has also been suggested that high [Na^+^]_c_ in heart failure can interfere with mitochondrial energy metabolism and decrease mitochondrial Ca^2+^ ([Ca^2+^]_m_) levels, further affecting the myocardial electrical activity and mechanical contraction (67). Thus, the effect of SGLT2i on myocardial [Na^+^]_c_ and the associated antiarrhythmic effects were not fully understood, and reports of NHE-1 activity and Na^+^ homeostasis were inconsistent and needed to be further explored.

#### The effect of SGLT2i on related ion channel proteins/receptors

Research had implied that diminished SERCA2a activity and leaky RyR increased diastolic [Ca^2+^]_c_ in the failing heart, and the aberrant expression of ion channel proteins, which was the major trigger of the occurrence of arrhythmia. Of note, SGLT2i may affect Ca^2+^ handling, Na^+^ balance and mitochondrial ROS released through to affect the ion channel proteins, which may have an antiarrhythmic effect. In a rodent study, dapagliflozin induced SERCA2a activity increase (68). Empagliflozin induced an increase in phosphorylated phospholamban and an improvement in SERCA2a function in a similar manner (46). Additionally, it was reported that empagliflozin was responsible for hyperphosphorylated RyR, which led to a gradual SR leak through the reduction of CaMK II activity (6, 69). It was notable that CaMK II upregulation plays a pivotal role in the pathogenesis of cardiovascular diseases (70), it was observed that long-term administration of canagliflozin significantly reduced ischemia/reperfusion injury on myocardial tissue in diabetic and non-diabetic rats, which was probably caused by a decline in CaMK II (71). Additionally, in failure hearts, NHE-1 was overexpressed, causing an accumulation of [Na^+^]_c_ and subsequent [Ca^2+^]_c_ overload, SGLT2i counteract those pathological processes by inhibition of NHE-1 (72).

### The effect of SGLT2i on cardiac electrophysiology

Electrical remodeling of the cardio can cause shortening and prolongation of the effective refractory period or uncoordinated conduction, simultaneously, structural remodeling causes electrical conduction delay or disorder (73). SGLT2i has a regulating and stabilizing effect on cardiac electrophysiological changes, which may be a potential mechanism by which SGLT2i exerts its antiarrhythmic effect. Research has shown that empagliflozin reduced late sodium channel current (late-INa) in cardiomyocytes in mice with heart failure or a sodium channel mutation, but not in healthy murine cardiac myocytes (74). A reduction in late-INa contributes to less prolongation of the cardiac action potential duration (APD) and may protect against arrhythmias associated with prolonged action potentials (64). Empagliflozin treatment significantly ameliorates sotalol-induced QTc prolongation in rats (75), empagliflozin also significantly reduced vulnerability to VA in rabbit hearts following ischemia-reperfusion (76). Dapagliflozin also improved mitochondrial function in rats with metabolic syndrome by enhancing insulin resistance, which inhibited ventricular repolarization (77). Thus, SGLT2i may inhibit arrhythmias by directly altering the electrophysiological characteristics of the diseased heart.

## Indirect mechanisms by which SGLT2i exerts anti-arrhythmic effects

To further explore the antiarrhythmic effect of SGLT2i, it is necessary to study its related indirect mechanisms of it, which mainly include reductions in cardiac load, improvement in heart failure, inhibition in sympathetic nerve activity, and reduction in body weight by SGLT2i, which can be considered indirect mechanisms.

### SGLT2i reduces the ventricular pressure load and volume load

Increasing blood pressure or myocardial oxygen consumption by any means may induce atrial or ventricular arrhythmias both experimentally and in patients, conversely, a decreasing in blood pressure or cardiac load (i.e., preload, afterload) may eliminate arrhythmias due to its causes. SGLT2i mainly acted on SGLT2 receptors in renal proximal tubular epithelial cells, inhibiting Na^+^ and glucose reabsorption, significantly increasing urine output, reducing cardiac preload and myocardial oxygen consumption, and lowering blood pressure (78). It had also been reported that SGLT2i improved the function of vascular endothelial cells and smooth muscle cells, and reduced vascular stiffness and vascular resistance (79). In addition, a reduction in total body Na^+^ had been reported to alleviate arterial stiffness, activating voltage-gated potassium channels and protein kinase G, causing vasodilation and further reducing the cardiac load (80). Reduced sympathetic activity and body weight loss were linked to reduced blood pressure (81). Even though all SGLT2i reduced blood pressure, based on an indirect meta-analysis, canagliflozin was found to cause a greater reduction of systolic blood pressure compared to other SGLT2i (82). Thus, SGLT2i may act as an antiarrhythmic effect through this indirect mechanism of lowering blood pressure or cardiac oxygen consumption.

### SGLT2i improve heart failure

Multiple trials had demonstrated the effect of SGLT2i to reduce overall mortality, particularly, patients with heart failure (2, 83, 84). As mentioned above, SGLT2i might reduce volume overload and improve cardiac function in heart failure patients (85). Researchers have reported that empagliflozin reduces blood pressure, arterial stiffness, and vascular resistance, improve the cardiac output of heart failure patients (86). Thus, the initial finding and the largest mechanism for the cardiac benefit of SGLT2i was its ameliorative effect on heart failure, which may also be its indirect anti-arrhythmic mechanism.

### SGLT2i inhibits sympathetic nerve activity

Cardiac sympathetic hyperinnervation and autonomic imbalance promote cardiac arrhythmias. Studies have shown that SGLT2i can inhibit sympathetic tone, reduce the secretion of sympathetically active substances in plasma and direct toxic effects on cardiomyocytes, and reduce myocardial oxygen consumption to protect and maintain normal cardiac function (87). SGLT2i can also reduce the expression of tyrosine hydroxylase in sympathetic nerves and decrease the secretion of norepinephrine, reducing the effect of sympathetic nerves on effector organs (25). In addition, SGLT2i can also regulate sympathetic activity through certain indirect mechanisms; for example, SGLT2i can inhibit sympathetic activity by reducing plasma leptin levels (88). In addition, SGLT2i can reduce sympathetic tension by decreasing neural activity in the vascular zone of the hypothalamic endplate through a natriuretic effect, which reduces the concentration of Na^+^ in the blood (89). Thus, this may also be another indirect mechanism by which SGLT2i exerts an antiarrhythmic effect.

### SGLT2i reduces body weight

Weight gain and obesity are closely related to arrhythmogenesis, and weight reduction is an essential component of arrhythmia intervention. SGLT2i achieve negative energy balance through diuresis, Na^+^ excretion, and glucose excretion, leading to weight loss. In obese rats, empagliflozin not only reduces body weight but also improves endothelial function and cardiac remodeling (90). Clinical studies had shown that SGLT2i significantly reduced body weight and suppressed obesity compared to placebo, which can result in a 2–3 kg weight loss, mainly by promoting osmotic diuresis leading to volume loss (91). Although SGLT2i-mediated weight loss was modest, its combination with a modest drop in preload and afterload could synergistically improve cardiac workload and contractility (92). Thus, this may also be a possible mechanism by which SGLT2i exerts an antiarrhythmic effect.

## Possible risks associated with the use of SGLT2i

Although SGLT2i reduced hospitalizations and adverse cardiovascular events among patients with heart failure, and were widely used clinically, possible complications associated with urinary excretion and hypoglycemia, such as urinary tract infections or ketoacidosis, and the associated off-target effects (drug side effects due to the action of the drug on additional targets) should not be overlooked. One study noted that some patients discontinued SGLT2i use after developing chronic or recurrent genital infections, and the remaining adverse events included polyuria, nausea, hypotension, dizziness, acute coronary events, deteriorations in glycaemic control status, and rapid deteriorations in renal function (93). It was also reported that the incidence of genital tract infections following SGLT2i treatment was 4.8%, however, women's rates were higher than men's; these were generally mild-to-moderate infections, and some patients were at risk of coinfection with fungal infections, but the benefits of SGLT2i application outweigh the disadvantages (94). In addition, there have been reports of increased risks of fracture and amputation with canagliflozin, the cause of which is thought to be related to reduced blood circulation (95), increased serum phosphate levels, and reduced vitamin D levels, and weight loss (96). It had also been reported that SGLT2i application increases the risk of diabetic ketoacidosis and ketonemia (97). This finding suggested that patients who were administered SGLT2i should be closely monitored for side effects. It is important, however, to consider the risks and benefits of SGLT2i before prescribing it. An in-depth study of the mechanisms of SGLT2i's beneficial effects as well as side effects or adverse effects was necessary.

## Conclusion and prospects

This review focuse on the antiarrhythmic effects of SGLT2i and the potential mechanisms. Due to the diversity of targets of SGLT2i-mediated cardioprotective effects, these agents can act directly or indirectly through cellular molecular mechanisms such as the downregulation of CaMK II activity, inhibition of NHE-1, repair of Ca^2+^ handling, stabilization of Na^+^ imbalances, and reduction in oxidative stress and indirectly by reducing cardiac load, improving myocardial energy metabolism, inhibiting inflammation, improving myocardial remodeling, inhibiting sympathetic nerve activity, reducing body weight and other organ function modifications to exert antiarrhythmic effects; however, the exact mechanism remains unclear, and there is no direct evidence of the antiarrhythmic effects of SGLT2i. Nevertheless, it is universally acknowledged that SGLT2i direct effects on the myocardium and systemic effects contribute to the cardioprotective effects of SGLT2i. Under the concept of “CARE ME” (cardio+renal+metabolic) comorbidity management for T2DM (98), SGLT2i are well-established in clinical practice, and relevant clinical studies and basic experiments have reported that SGLT2i have good antiarrhythmic effects, however, the exact mechanisms of still need to be further investigated.

## Author contributions

JW and XS designed the study. JW wrote the manuscript. YL, XZ, XW, and WL reviewed and edited the manuscript. YY and XS contributed to the discussion. All authors read, approved the final manuscript, and approved the final version to be published.

## Funding

This work was supported by the application and basic research project from the science and technology department of Qinghai province (Grant No. 2022-ZJ-758).

## Conflict of interest

The authors declare that the research was conducted in the absence of any commercial or financial relationships that could be construed as a potential conflict of interest.

## Publisher's note

All claims expressed in this article are solely those of the authors and do not necessarily represent those of their affiliated organizations, or those of the publisher, the editors and the reviewers. Any product that may be evaluated in this article, or claim that may be made by its manufacturer, is not guaranteed or endorsed by the publisher.
